# Equine Infectious Anaemia: The Active Surveillance of an Entire Equid Population Reduces the Occurrence of the Infection

**DOI:** 10.1155/2024/3439871

**Published:** 2024-04-30

**Authors:** Andrea Carvelli, Roberto Nardini, Azzurra Carnio, Ida Ricci, Francesca Rosone, Marcello Sala, Sara Simeoni, Daniela Maccarone, Maria Teresa Scicluna

**Affiliations:** ^1^Epidemiology Unit, Istituto Zooprofilattico Sperimentale del Lazio e della Toscana, M. Aleandri, via Appia Nuova 1411, 00178, Rome, Italy; ^2^WOAH Reference Laboratory for Equine Infectious Anaemia, Italian Reference Centre for Equine Infectious Anaemia, Istituto Zooprofilattico Sperimentale del Lazio e della Toscana, M. Aleandri, via Appia Nuova 1411, 00178, Rome, Italy

## Abstract

Equine infectious anaemia (EIA) is a life-long viral infection affecting equids, transmitted mechanically by biting flies and iatrogenic means. Despite its global distribution, active surveillance is limited, with passive clinical surveillance or control of specific equine sectors prevailing. In Italy, a national surveillance plan in horse, donkey, and mule populations has been established and includes mandatory passive and active surveillance through annual serological tests. During 2007–2010, the agar gel immunodiffusion (AGID) test served as both screening and confirmatory tests. Since 2011, a three-tier diagnostic pathway was introduced, utilizing the ELISA test for screening, AGID as the confirmatory test, and the immunoblot test for cases where ELISA was positive and AGID was negative. From a total equid population of 406,000 animals, 1,337,899 samples were analysed during 2007–2012, with 2,348 (0.18%) testing positive. EIA seroprevalence significantly decreased across all the species/hybrids during the study period. EIA occurrence was higher in mules (IRR = 48.90) and lower in donkeys (IRR = 0.56) compared to horses. The holding seroprevalence was 1.15%. Spatial analysis revealed clusters of infection in central Italy. These findings demonstrate that active systematic surveillance effectively reduces EIA prevalence in equid populations. Mules and working horses in wooded areas appeared to be at higher risk of infection and act as EIA reservoirs. Surveillance and control should be maintained and strengthened in these species/hybrids and in these areas to effectively control EIA. Passive surveillance alone is insufficient to eradicate the disease, and EIA remains a constant threat for the equine industry if active control is not implemented.

## 1. Introduction

Equine infectious anaemia (EIA) is a persistent infectious disease caused by a virus belonging to the Retroviridae family, genus *Lentivirus*, affecting equids (horses, donkeys, mules, hinnies, and zebras) [1]. The infection is mechanically transmitted from an infected to a susceptible animal through the bite of haematophagous vectors (such as horse flies *Tabanus* spp., deer fly *Chrysops* spp., and stable fly *Stomoxys calcitrans*), although contaminated medical equipment and blood-based products are considered the most important modes of transmission [2, 3]. Aerosol and venereal transmissions have also been hypothesised as a source of infection [4]. The disease can occur in three clinical forms: acute, subacute, and chronic, with the latter being the most frequent. In the chronic form, stress factors impacting the immune system (overworking, other diseases, pregnancy, malnutrition, and corticosteroid therapy) can cause the recrudescence of EIA clinical signs (fever, lethargy, inappetence, thrombocytopenia, and anaemia) and recurrent viremia [5]. Possible intermittent acute episodes may lead to the death of the animal or the status of unapparent carrier [6]. After infection, animals remain persistently infected and can be contagious during viremic phases. To date, there is no effective treatment or vaccine available. EIA has a significant impact on international trade in countries where the horse industry is relevant.

Most epidemiological and experimental studies are usually carried out on horses, but the virus also infects donkeys and their hybrids, i.e., mules and hinnies. Although it is generally accepted that mules are likely to develop subclinical infection, there is limited literature concerning their epidemiological importance in the maintenance and transmission of EIA virus (EIAV). Naturally infected mules can present a viremic load as a consequence to immunosuppression, acting as an EIAV reservoir even in the chronic form of the disease [7, 8]. Furthermore, infected mules with a positive enzyme-linked immunosorbent assay (ELISA) and an equivocal or negative agar gel immunodiffusion (AGID) test were found to have the same level of viral load of animals with a high antibody titre, suggesting a potential role of mules as an EIAV reservoir independently of the serological pattern [8, 9]. Few studies reported EIA occurrence in mules and donkeys [10], and available data can be biased by the difficulty in interpreting the AGID test, which can produce false negative results [9, 11]. In mules, the prevalence reported in the literature ranged from 0% (0/51, Ethiopia) to 3.5% (27/767, central Italy), while in donkeys, it varied from 0% (0/1568; central Italy), 0.2% (1/662; Ethiopia), and 3.3% (12/367; Brazil) to 8.3% (14/169; Sudan) [10].

EIA is a notifiable disease worldwide with a global distribution [1], but in many countries, only passive clinical surveillance and mitigation measures, as prescribed by the World Organisation for Animal Health (WOAH), are applied [12, 13]. Since most infections are subclinical, the lack of routine active surveillance for EIA leads to underdiagnoses/underreporting. Active surveillance plans, including systematic periodic testing of whole equid population, are officially ongoing in few countries worldwide, such as Italy, Romania, and Serbia, since 2007, 1996, and 1981, respectively [14–16], and in India from 1999 to 2012 [17]. No exhaustive studies describing spatial pattern, incidence, prevalence, and temporal trends are available. Most of the published papers investigating EIA epidemiology originated from a clinical index case, from epidemiological investigations following passive surveillance, and from active surveillance studies with a sampling design not representative of the national equid population [2, 18–24]. Only a few studies were conducted with a proper design and a representative sampling aimed at estimating the occurrence of the infection in a region, a country, an equine sector, or the whole equid population [12, 25–29]. Therefore, extensive variation in EIA occurrence is found worldwide, depending on the population, study area, and design. Some countries reported a disease-free status, while others reported a seroprevalence range from 0.4% to 56% [21–23, 29, 30].

In Europe, no comprehensive studies on EIA occurrence are available, but outbreaks are regularly reported (WAHIS, WOAH). In the European Union (EU), according to Regulation (EU) 2016/429 and Commission Implementing Regulation (EU) 2018/1882, EIA belongs to category D (diseases for which measures are needed to prevent them from spreading on account of their entry into the EU or movements between member states) and category E (diseases for which there is a need for surveillance within the EU). Without a standardised approach, surveillance and control activities differ significantly among European countries. Romania and Italy are often considered endemic [13, 14], but in these two countries, a surveillance plan is in place with systematic testing of the whole equid population. In other European countries, where only passive clinical surveillance or surveillance in a few sectors (breeding stallions, sport horses, and horses for export) is in place, the infection is reported with a sporadic trend [14]. In countries where EIA re-emerged, such as Belgium, Germany, Ireland, and Great Britain, epidemiological studies suggested that equine biological products (blood plasma) or infected imported animals were the source of the infection [2, 14, 18]. During the last decade, as reported in WOAH WAHIS, EIA outbreaks were notified in Austria, Belgium, France, Germany, Great Britain, Greece, Spain, Switzerland, and the Netherlands.

In Italy, EIA has been a notifiable disease since 1954 [31, 32]. During 1995–2006, EIA control using the AGID test was applied only in case of horse movement and to breeding stallions (Directive 90/426/EEC). A comprehensive EIA surveillance plan of the entire equid population, called the EIA National Surveillance Plan (NSP), started in 2007 and ended in 2012, when risk-based surveillance was applied at the national level [15, 16, 33]. The objectives of the NSP were to eradicate EIA, to assess temporal and spatial trends, to adopt control measures, and to identify EIA risk factors and clusters.

The aim of this study is to report the findings of the EIA active surveillance plan in horses, donkeys, and mules, in Italy, during 2007–2012 and to demonstrate that active surveillance of the whole equid population reduces the occurrence of the infection.

## 2. Materials and Methods

### 2.1. EIA National Surveillance Plan

The Italian EIA NSP started in 2007 [16], including both passive and active surveillance. Private practitioners were obliged to notify suspected clinical cases to the local health unit (LHU) and collect blood samples to submit for EIA detection to the laboratory network of Istituti Zooprofilattici Sperimentali (IZS). Active surveillance consisted of a annual serological testing of the entire national equid population (horses, donkeys, mules, hinnies, and zebras) over 6 months of age. Horses bred for meat production were excluded from sampling as they were considered at minimal risk for spreading the infection due to their short lifespans and clear separation from other horse sectors. During 2011–2012, considering the spatial pattern of the EIA outbreaks detected since 2007, the frequency of sampling remained annual in four central regions (Abruzzo, Lazio, Umbria, and Molise) and in holdings with at least one mule, regardless of the geographic zone, while in the remaining regions, the frequency of the EIA serological test was reduced, with testing prescribed every 2 years.

Blood samples were collected by public and private practitioners appointed by LHU and analysed by IZS laboratories. Screening test results were transmitted to the EIA National Reference Centre (EIA NRC) along with a set of metadata (Table 1). Samples resulting positive to the screening test were sent for confirmation to the EIA NRC. LHU notified suspect or confirmed outbreak in the National Animal Disease Notification System (Siman: Sistema Informativo Malattie Animali Nazionale) and applied control measures. A suspect case had to be isolated, and movement restrictions were applied until the result of the confirmatory test. In case of a confirmed positive test, culling or permanent quarantine was carried out. The latter consists of life-long isolation (at least 200 m away from other equids) to prevent the spread of the infection, compulsory use of insect-proof nets in stable windows and doors, periodic insecticide use and disinfection, and insect traps to test. Movements from infected holdings were banned, and trace-back and trace-forward movements and testing of contact animals were performed [15, 16, 33].

### 2.2. Laboratory Tests Pathway

In the period 2007–2010, the AGID test was used as the screening test and also as the confirmatory test, as described by the WOAH Manual of Diagnostic Tests and Vaccines for Terrestrial Animals in force at that time [34]. From 2011, the diagnostic pathway was changed to enhance the sensitivity and the specificity of the surveillance system [11] into a three-tier system. A serological ELISA was performed as the screening test by the IZS laboratories, and AGID was used as the confirmatory assay, performed by the EIA NRC, according to the WOAH Manual of Diagnostic Tests and Vaccines for Terrestrial Animals. When an ELISA-positive test was confirmed with an AGID-positive result, the sample was confirmed as positive. When ELISA resulted positive or equivocal and AGID negative, an immunoblot assay (IB) was used as an alternative confirmatory assay [9, 35]. The complex-trapping blocking ELISA developed by the EIA NRC was a competitive ELISA that employed a p26 recombinant antigen and two monoclonal antibodies (MABs) (catcher and tracer), respectively, for adsorbing the antigen to the ELISA plates and for detecting the formation of the MAB–antigen complex. Other ELISAs were also available both commercial and produced by another IZS laboratory. The latter employed the same antigen and MABs already described, while the commercial kits employed a modified sandwich ELISA with a purified recombinant p26 antigen (VMRD®, Pullman, USA), a double antigen ELISA with a recombinant p26 antigen (IDVET®, France), and an indirect ELISA using recombinant *gag*- and *env*-derived antigens. The IB test investigated the presence of serum antibodies against three EIAV antigens : protein p26, glycoprotein (gp) 45, and gp 90. A sample was considered IB positive when presenting a reaction for at least p26 and one of the other antigens. Different patterns were considered negative. As several commercial ELISA tests were available, the EIA NRC validated two ELISA tests and one IB test, according to WOAH guidelines [35–38], and performed test evaluation, with good concordance among the different assays. The diagnostic performances of the IZS laboratories were monitored by inter-laboratory trials, organised by the EIA NRC every 2 years [39].

### 2.3. Data Analysis

The study takes into consideration EIA surveillance data for the 2007–2012 period. Descriptive and statistical analyses were conducted at animal level and holding level.

Annual seroprevalence were calculated for horses, mules, and donkeys. A positive sample was defined as an equid tested positive at screening (ELISA or AGID positive test by IZS) and confirmed in AGID or IB by the EIA NRC.

The EIA seroprevalence temporal trend was estimated by a Poisson regression model using 2007 and horses as baseline values. The incidence rate ratio (IRR) and the 95% confidence interval (95% CI) were calculated using Stata 16.0 (StataCorp. 2019).

At holding level, the annual seroprevalence of positive holdings was calculated, considering a positive holding as a holding with at least one EIA confirmed case. The Italian holdings with equids were obtained from the National Animal Registry Database (Banca Dati Nazionale, BDN).

Cluster analysis of EIA outbreaks during 2007–2012 was performed with the optimised hot spot analysis on ArcGis Pro^©^ software (ESRI Release 3.1). The distance band (threshold distance), estimated using the incremental spatial autocorrelation tool, was set at 130 km.

## 3. Results and Discussion

### 3.1. Animal Level

According to FAOSTAT, the Italian equid population remained stable and consisted of approximately 406,000 animals during the study period, including 373,000 horses, 24,000 donkeys, and 9,000 mules and hinnies (https://www.fao.org/faostat).

**Figure 1:**
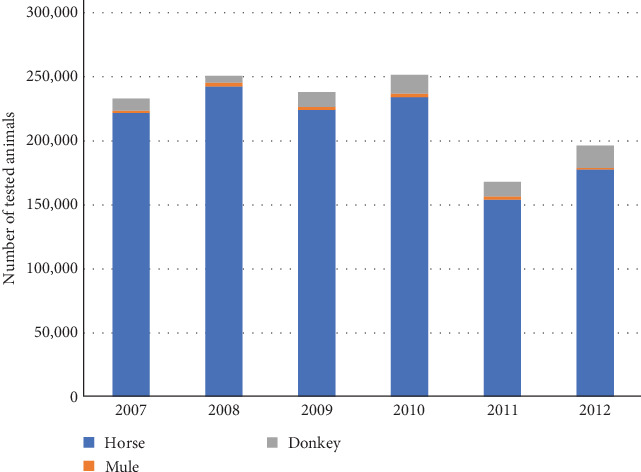
Number of EIA-tested samples during 2007–2012 per species/hybrids.

Throughout the study period, IZS laboratories analysed a total of 1,337,899 samples. The EIA NRC confirmed 2,348 (0.18%) positive samples. The distribution of samples analysed per species was as follows: horses (94%), mules (1%), and donkeys (5%), as illustrated in Figure 1.

**Figure 2:**
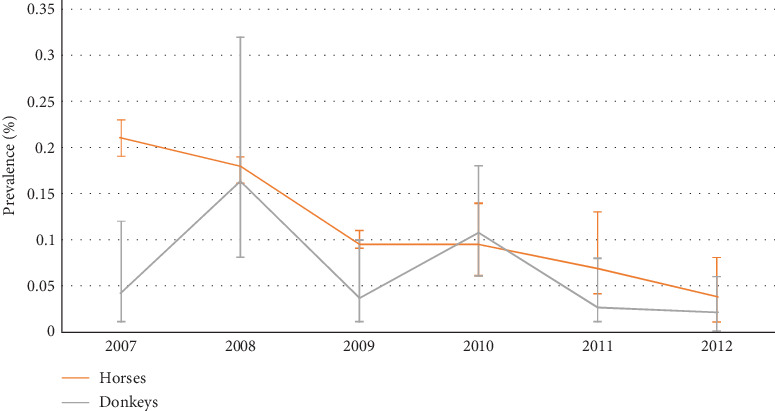
EIA seroprevalence and 95% confidence interval in horses and donkeys.

**Figure 3:**
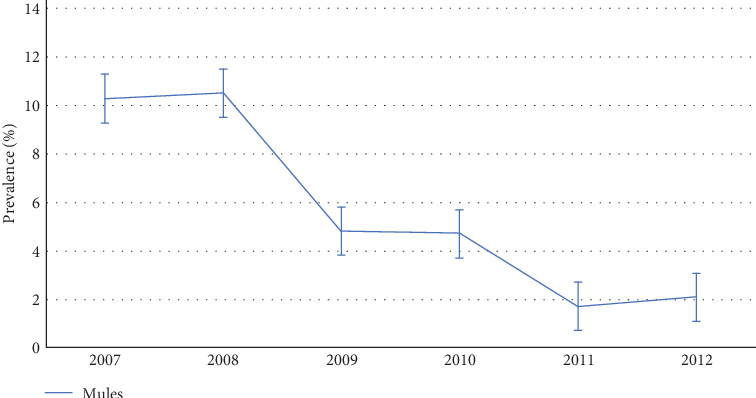
EIA seroprevalence and 95% confidence interval in mules.

The EIA-tested samples, seroprevalence, and confidence intervals are reported in Table 2 and Figures 2 and 3.

### 3.2. Horses

During 2007–2012, a total of 1,255,154 horse samples were analysed, with the EIA NRC confirming 1,546 (0.12%) positive samples. In 2011 and 2012, a reduction in tested samples occurred compared to previous years.

The average coverage of the entire horse population, calculated as the mean of sampled animals out of the total population, was 56%.

The seroprevalence of positive samples decreased from 0.21% (95% CI: 0.19–0.23) in 2007 to 0.04% (95% CI: 0.01–0.08) in 2012 (Figure 2).

### 3.3. Mules

During 2007–2012, a total of 13,078 mule samples were analysed, with the EIA NRC confirming 763 (5.83%) positive samples. A large reduction in tested samples occurred in 2012 compared to previous years.

The average coverage of the entire mule population, calculated as the mean of sampled animals out of the total population, was 48%.

The seroprevalence of positive samples changed from 10.27% (95% CI: 8.9–11.7) in 2007 to 2.08% (95% CI: 1.40–3.05) in 2012 (Figure 3).

### 3.4. Donkeys

During 2007–2012, a total of 69,667 donkey samples were analysed, with the EIA NRC confirming 39 (0.06%) positive samples. The average coverage of the entire donkey population, calculated as the mean of sampled animals out of the total population, was 56%.

The number of samples tested varied, showing an increasing trend.

The seroprevalence of positive samples was variable and remained at a low level (Figure 2).

### 3.5. Annual Trend of EIA Seroprevalence and Risk in Equid Species/Hybrids

The EIA seroprevalence decreased in all the species/hybrids during the study period, confirmed by a Poisson regression, which was significant from 2009 to 2012 (*p* ≤ 0.001).

A progressive IRR reduction was observed, particularly in 2011 (IRR = 0.25) and 2012 (IRR = 0.18). The regression indicated a higher occurrence of infection in mules (*p* ≤ 0.001; IRR = 48.90; 95% CI: 44.87–53.41) and a lower occurrence in donkeys (*p*  ≤ 0.001; IRR = 0.56; 95% CI: 0.41–0.77) compared to horses.

### 3.6. EIA Seroprevalence at Holding Level

During the period 2007–2012, 94,129 equid holdings out of 123,053 (BDN) were tested (94,129/123,053 = 76.5%) and 1,086 holdings with at least one confirmed case were recorded, and official outbreaks were notified. The cumulative seroprevalence was 1.15% (95% CI: 1.09–1.23) (Table 3).

### 3.7. Spatial Distribution

**Figure 4:**
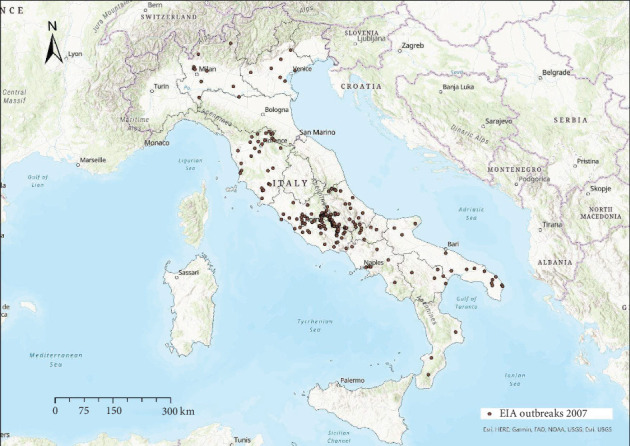
Location of EIA outbreaks 2007.

**Figure 5:**
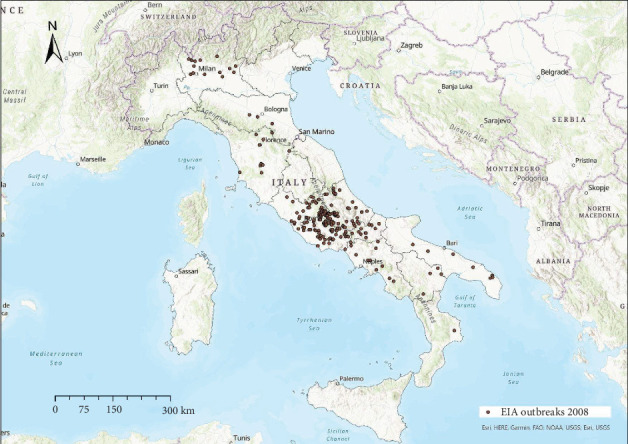
Location of EIA outbreaks 2008.

**Figure 6:**
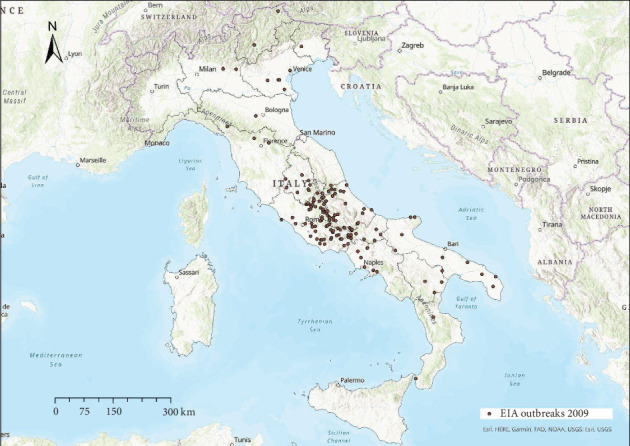
Location of EIA outbreaks 2009.

**Figure 7:**
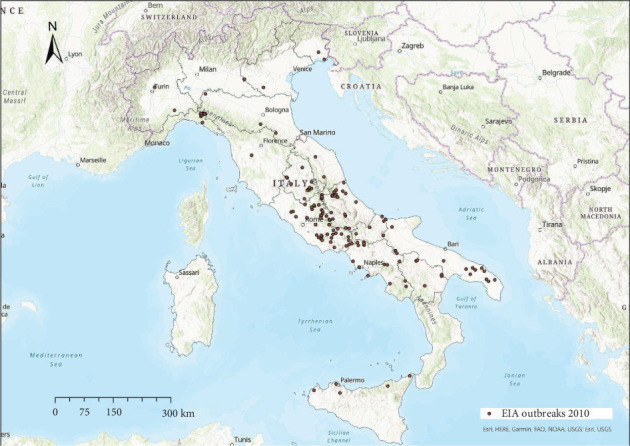
Location of EIA outbreaks 2010.

**Figure 8:**
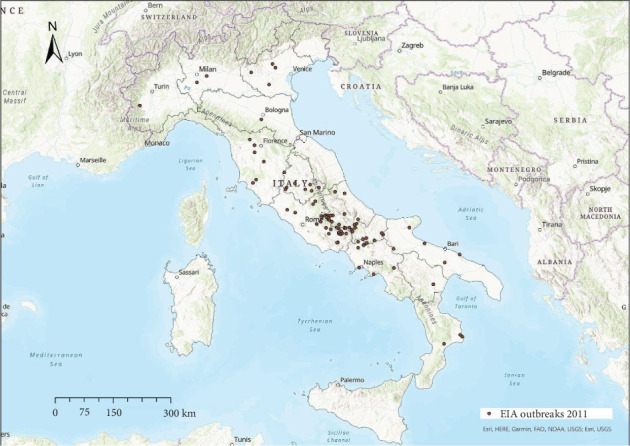
Location of EIA outbreaks 2011.

**Figure 9:**
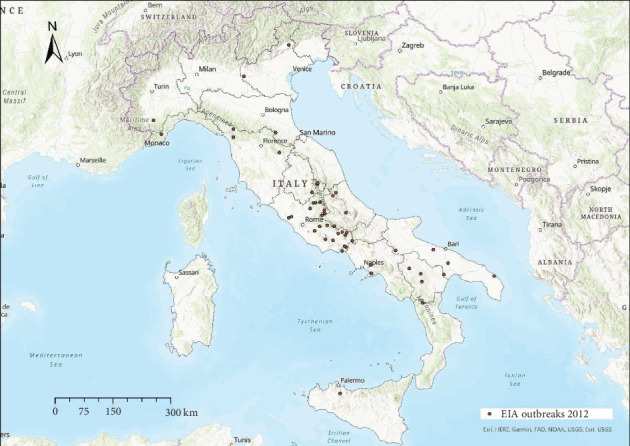
Location of EIA outbreaks 2012.

**Figure 10:**
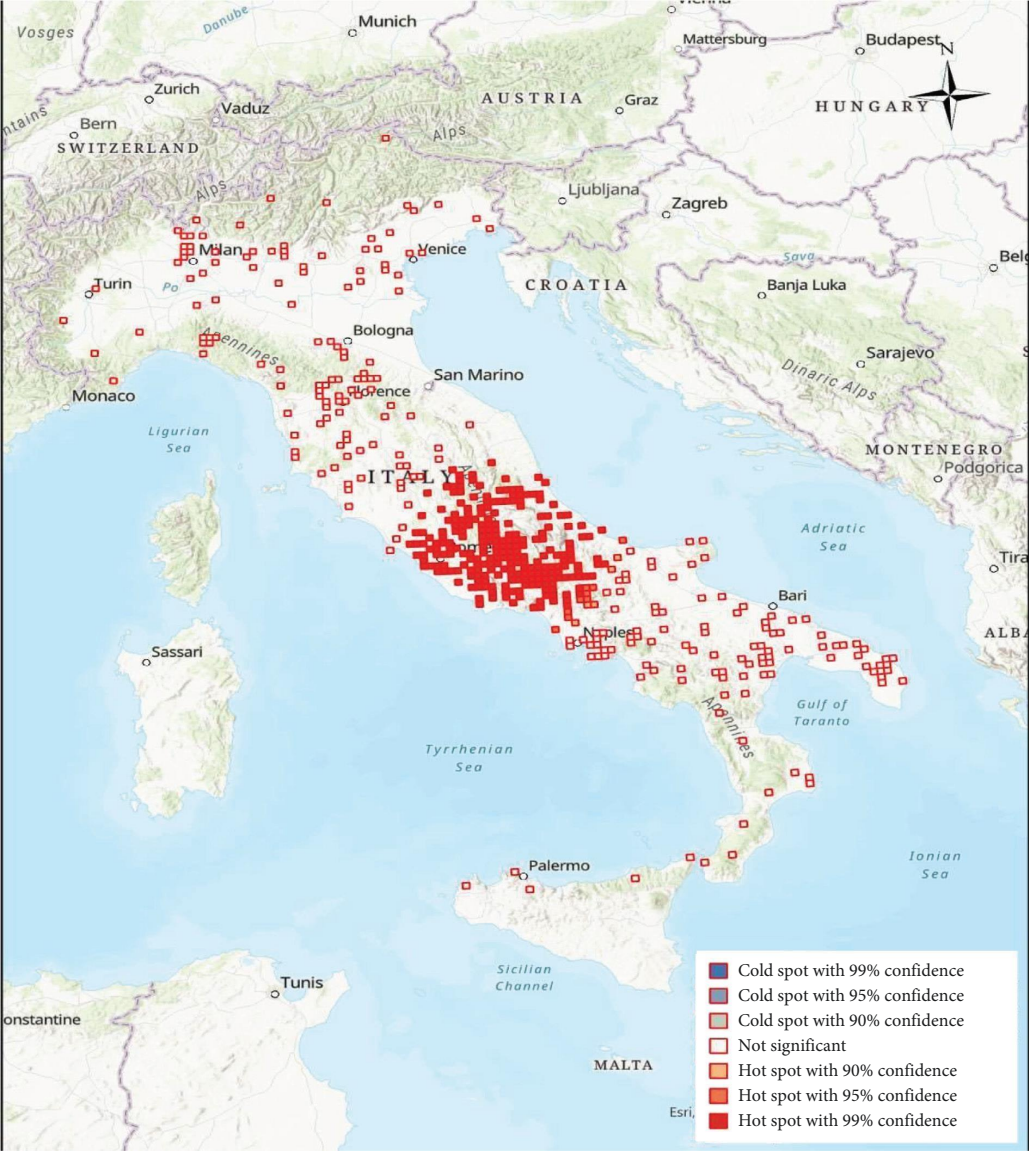
Hot spots of 2007–2012 EIA outbreaks in cluster analysis.

EIA outbreaks were mainly distributed in central Italy every year, with sporadic occurrence in other areas (Figures 4−10). Clustering of EIA outbreaks in central Italy was significant at a 99% confidence level (*p*-value < 0.01; *z*-score: 2.82–4.16) (Figure 10).

## 4. Discussion

This paper presents the first comprehensive epidemiological study of a nationwide structured EIA surveillance over a 6 years period covering the entire equid population. A significant reduction in the EIA prevalence was demonstrated during the study period across all three equid species/hybrids and related outbreaks.

EIA is rarely subject to structured national surveillance plans worldwide, similar to other equine infectious diseases like influenza and herpesviruses [40]. Apart from Italy, only two other countries, Romania and Serbia, have implemented similar surveillance levels. Without an active surveillance system, the absence of disease notification does not ensure the absence of the disease [14]. Given the life-long and often subclinical nature of the infection, passive (clinical) surveillance alone is insufficient for effective EIA eradication policies, posing a constant threat to the equine industry and trade.

The findings of this study demonstrate that active systematic surveillance over several years effectively reduce EIA prevalence. Poisson's regression revealed a notable reduction in prevalence, in 2011 and 2012, supporting the adoption of a risk-based surveillance strategy, i.e., one test a year in higher prevalence regions and one test every 2 years in lower prevalence regions. This approach indeed resulted in fewer samples collected and analysed (Figure 1 and Table 2), optimising economic resources while maintaining similar surveillance sensitivity, i.e., the same amount of positive cases (Table 2). Despite a 45% reduction in analysed samples compared to the previous year, the prevalence in mules increased from 1.72 to 2.08 in 2012, further validating the effectiveness of the approach (Table 2).

EIA prevalence varied consistently among horses, mules, and donkeys. Prevalence was consistently below 0.25% in horses and donkeys, while in mules, it ranged between 1.7% and 10.5%, confirming these hybrids are at major risk of being infected and represent an important reservoir [41]. In Italy, mules are mainly bred for recreational and agricultural purposes, particularly in rural and mountainous areas of central Italy. Mules are used to transport materials, especially wood in dense forests, where the use of mechanical means is not possible. In this environment, EIA competent vectors are abundant, mules are susceptible to the stress-related reactivation of viremia due to overworking or poor health conditions, and biosecurity measures are often inadequately implemented. Furthermore, in these marginal areas, the application of surveillance and control measures by the official veterinarians can be more difficult. Authors report that owners of the mules were often not willing to comply with the regulation in force. Despite significant decreases in seroprevalence in 2009 and 2011, mules remain a relevant reservoir, necessitating continued and reinforced surveillance and control measures in these areas.

The surveillance data for donkeys suggest lower intensity of sampling comparing to mules and horses, with the surveillance target not been achieved in different years. The denominators (i.e. population) indeed resulted inhomogeneous, leading to biased seroprevalence estimation.

Despite mandatory annual testing, the sampling coverage of the three equid populations was never close to 100%. This can be due to the incomplete animal and holding registration in BDN but also to the difficulty in accessing equids held privately, especially those never moved for breeding or sport events.

The BDN did not often provide information on the horse category during the considered years under consideration, but annual reports and epidemiological investigations by the EIA NRC reported that the equestrian, trotting, and jockey sectors were almost completely free from the infection, while EIA clusters were recorded in working horses and mules and in horses living with mules.

Regarding the spatial pattern, sporadic trends of EIA were observed in northern and southern Italy, while the clustering in central Italy remained consistent throughout the study period (Figures 4−10), with more than half of the EIA outbreaks notified in mountainous areas of Abruzzo, Lazio, Umbria, and Molise. Here, the use of horses and mules as working animals is still common, and animals are often not stabled indoors and more likely exposed to vectors. The presence of EIA-positive animals and inappropriate biosecurity measures remain persistent risk factors. In these areas, a positive correlation was found between EIA prevalence, tabanid abundance, and species diversity [42].

Furthermore, limited culling of positive equids during the study period occurred due to increased humane sensitivity and ethical reasons which increased the risk of EIA spread, highlighting the importance of maintaining a permanent, life-long, and strict application of the quarantine biosecurity measures.

The diagnostic sensitivity and specificity are crucial for the effective surveillance system aiming at eradication. The ELISA used as a screening test had a sensitivity of 100% (95% CI: 91.6–100.0) and a specificity of 73.2% (95% CI: 67.6–78.1) [36]. To increase specificity and reduce the number of false positive results, the three-tier diagnostic flow was introduced, with IB as the final discriminatory test, achieving almost 100% specificity in all species/hybrids, confirming the effectiveness of the diagnostic approach [35]. During the first 4 years of the surveillance activities considered in this study, the use of the sole AGID as a confirmatory test led to an expected but not negligible false negative animals, underestimating the seroprevalence. In view of this, the reduction in EIA occurrence at the national level can be considered higher than the reported decrease.

A limit of the present study is the incompleteness of the BDN in terms of registered holdings and animals. The BDN had been established few years before, and therefore, not all the equid holdings and animals were likely to be registered, leading to a potential overestimation of EIA holding prevalence. For the same reason, samples rather than animals were considered to calculate seroprevalence, but it is possible to assume that each animal was tested only once a year, according to the regulations in force. Furthermore, the absence of information in BDN precluded the assessment of possible association among EIA and potential risk factors such as age, sex, attitude, and other individual and holding factors.

Another limitation is the time period considered, dating back a decade ago. In the years following this study, 2013–2023, the epidemiological situation of EIA remained fairly constant. The number of outbreaks was low, with a sporadic trend, and the spatial cluster remained localised in central Italy. These and further details, such as risk factors for EIA infection, during the period 2013–2023, will be discussed in another article.

## 5. Conclusions

In conclusion, the intensive 6 years of EIA active surveillance in the Italian equid population and the control measures applied for positive cases reduced EIA presence in Italy across all the species/hybrids. Mules represent a higher risk of infection and a major threat to the spread of EIA. Outbreaks are clustered in central Italy, probably due to the use of working equids in mountainous areas, necessitating focused surveillance in this region/sector. The economic impact of consistent surveillance activities should be investigated through further cost-benefit studies. Finally, this study enabled evaluation of EIA risk factors, informing dynamic evaluation and revision of surveillance activities, critical components of health systems.

## Figures and Tables

**Table 1:** Data collected on EIA surveillance activities.

Section	Data
Administrative unit	LHU, municipality, province, region

Purpose of sampling	Serological surveillance
	Clinical surveillance
	Control of susceptible animal after EIA-positive case removal
	Animal trade

Date	Sampling date

Epidemiological unit (holding)	Holding name
	Holding ID code
	Owner name and fiscal code
	Owner data address
	Holding category: stud farm, trotting breeding centre, jockey breeding centre, saddle breeding centre, riding school, race track, meat production, trotting training centre, jockey training centre, saddle training centre, other
	Animal category: equestrian with/without breeders, jockey with/without breeders, trotting with/without breeders, meat with/without breeders, working/draft
	Geographical coordinates (WGS84)

Animal data	Microchip (or passport ID)
	Species
	Breed
	Sex
	Date of birth
	Owner name

**Table 2:** EIA-tested samples, coverage of the whole population, seroprevalence, and related confidence intervals per species/hybrids.

Species/hybrids	Year	Tested samples (*N*)	Coverage (%)	Seroprevalence (%)	95% CIs	
					Lower	Upper
Horses	2007	221,916	59	0.21	0.19	0.23
	2008	242,806	65	0.18	0.16	0.19
	2009	224,318	60	0.10	0.09	0.11
	2010	234,259	63	0.10	0.06	0.14
	2011	154,165	41	0.07	0.04	0.13
	2012	177,690	48	0.04	0.01	0.08

Mules	2007	1,772	20	10.27	8.85	11.68
	2008	2,634	29	10.52	9.38	11.77
	2009	2,202	24	4.81	3.98	5.81
	2010	2,784	31	4.70	3.93	5.54
	2011	2,387	27	1.72	1.32	2.44
	2012	1,299	14	2.08	1.40	3.05

Donkeys	2007	9,376	39	0.04	0.01	0.12
	2008	5,463	23	0.16	0.08	0.32
	2009	11,532	48	0.03	0.01	0.10
	2010	14,695	61	0.11	0.06	0.18
	2011	11,324	47	0.03	0.01	0.08
	2012	17,277	72	0.02	0.00	0.06

**Table 3:** EIA-tested holdings, coverage of the whole population, number of outbreaks, seroprevalence, and seroprevalence 95% confidence intervals (CIs).

Year	Tested holdings (*N*; coverage %)	No. of outbreaks	Seroprevalence (%)	95% CIs	
				Lower	Upper
2007	46,166 (37.5)	356	0.77	0.61	1.00
2008	48,586 (39.5)	334	0.69	0.53	0.87
2009	45,702 (37.1)	145	0.32	0.21	0.45
2010	49,121 (39.9)	132	0.27	0.18	0.39
2011	37,773 (30.7)	76	0.20	0.12	0.35
2012	40,920 (33.3)	43	0.11	0.05	0.14
Total	94,129 (76.5)	1,086	1.15	1.09	1.23

## Data Availability

Data are not available because considered sensitive as they contain the animal owner's name and the location of the farm. Data are available upon request to andrea.carvelli@izslt.it.

## References

[1] WOAH (2023). *Manual of Diagnostic Tests and Vaccines for Terrestrial Animals*.

[2] More S. J., Aznar I., Bailey D. C. (2008). An outbreak of equine infectious anaemia in Ireland during 2006: investigation methodology, initial source of infection, diagnosis and clinical presentation, modes of transmission and spread in the Meath cluster. *Equine Veterinary Journal*.

[3] Williams D. L., Issel C. J., Steelman C. D., Adams W. V., Benton C. V. (1981). Studies with equine infectious anemia virus: transmission attempts by mosquitoes and survival of virus on vector mouthparts and hypodermic needles, and in mosquito tissue culture. *American Journal of Veterinary Research*.

[4] More S. J., Aznar I., Myers T., Leadon D. P., Clegg T. A. (2008). An outbreak of equine infectious anaemia in Ireland during 2006: the modes of transmission and spread in the Kildare cluster. *Equine Veterinary Journal*.

[5] Kono Y., Hirasawa K., Fukunaga Y., Taniguchi T. (1976). Recrudescence of equine infectious anemia by treatment with immunosuppressive drugs. *National Institute of Animal Health quarterly*.

[6] Cheevers W. P., McGuire T. C. (1985). Equine infectious anemia virus: immunopathogenesis and persistence. *Clinical Infectious Diseases*.

[7] Autorino G. L., Eleni C., Frontoso R. (2016). Evolution of clinical, virological and histological findings of equine infectious anaemia (EIA) in naturally infected mules following immune suppression. *Journal of Equine Veterinary Science*.

[8] Autorino G. L., Eleni C., Manna G. (2016). Evolution of equine infectious anaemia in naturally infected mules with different serological reactivity patterns prior and after immune suppression. *Veterinary Microbiology*.

[9] Scicluna M. T., Issel C. J., Cook F. R. (2013). Is a diagnostic system based exclusively on agar gel immunodiffusion adequate for controlling the spread of equine infectious anaemia?. *Veterinary Microbiology*.

[10] Câmara R. J. F., Bueno B. L., Resende C. F., Balasuriya U. B. R., Sakamoto S. M., Reis J. K. P. (2020). Viral diseases that affect donkeys and mules. *Animals*.

[11] Issel C. J., Scicluna M. T., Cook S. J. (2013). Challenges and proposed solutions for more accurate serological diagnosis of equine infectious anaemia. *Veterinary Record*.

[12] Higgins S. N., Howden K. J., James C. R., Epp T., Lohmann K. L. (2017). A retrospective study of owner-requested testing as surveillance for equine infectious anemia in Canada (2009–2012. *Canadian Veterinary Journal-Revue Veterinaire Canadienne*.

[13] Roberts H. (2017). Equine infectious anaemia in Europe: an ongoing threat to the UK. *Veterinary Record*.

[14] Bolfa P., Barbuceanu F., Leau S.-E., Leroux C. (2016). Equine infectious anaemia in Europe: time to re-examine the efficacy of monitoring and control protocols?. *Equine Veterinary Journal*.

[15] Ministero della Salute (2006). Disposizioni urgenti in materia di sorveglianza dell’anemia infettiva degli equidi.

[16] Ministero della Salute (2007). Piano di sorveglianza nazionale per l’anemia infettiva degli equidi.

[17] Malik P., Singha H., Goyal S. K. (2013). Sero-surveillance of equine infectious anemia virus in equines in India during more than a decade (1999–2012). *Indian Journal of Virology*.

[18] Caij A. B., Tignon M. (2014). Epidemiology and genetic characterization of equine infectious anaemia virus strains isolated in Belgium in 2010. *Transboundary and Emerging Diseases*.

[19] Zambruno T., Elgue F., Lauricica P., Costa A. L. (2016). Equine infectious anemia cases in polo ponies housed in San Isidro’s training center, Buenos Aires, Argentina. *Journal of Equine Veterinary Science*.

[20] De Queiroz Baptista D., Bruhn F. R. P., Da Rocha C. M. B. M. (2016). Temporal series analyses in equine infectious anemia cases in the State of Rio de Janeiro, Brazil, 2007 to 2011. *Revista Brasileira De Medicina Veterinaria*.

[21] Albayrak H., Ozan E. (2010). Serosurveillance for equine infectious anaemia in the Ardahan province of Turkey. *Tropical Animal Health and Production*.

[22] Alnaeem A. A., Hemida M. G. (2019). Surveillance of the equine infectious anemia virus in eastern and central Saudi Arabia during 2014–2016. *Veterinary World*.

[23] Talafha A. Q., Abutarbush S. M., Rutley D. L. (2016). Epidemiologic status of equine viral arteritis, equine infectious anemia, and glanders in Jordan. *Journal of Equine Veterinary Science*.

[24] Cursino A. E., Lima M. T., Furlan Nogueira M. (2021). Identification of large genetic variations in the equine infectious anemia virus tat-gag genomic region. *Transboundary and Emerging Diseases*.

[25] Pagamjav O., Kobayashi K., Murakami H. (2011). Serological survey of equine viral diseases in Mongolia. *Microbiology and Immunology*.

[26] Barros M. L., Borges A. M. C. M., Oliveira D. E., Lacerda W., Souza D. E. O., Aguiar D. M. (2018). Spatial distribution and risk factors for equine infectious anaemia in the state of Mato Grosso, Brazil. *Revue Scientifique et Technique de l’OIE*.

[27] Lupulovic D., Savić S., Gaudaire D. (2021). Identification and genetic characterization of equine infectious anemia virus in Western Balkans. *BMC Veterinary Research*.

[28] Borges A. M. C. M., Silva L. G., Nogueira M. F. (2013). Prevalence and risk factors for equine infectious anemia in Poconé municipality, northern Brazilian Pantanal. *Research in Veterinary Science*.

[29] Cruz F., Fores P., Ireland J., Moreno M. A., Newton R. (2015). Freedom from equine infectious anaemia virus infection in Spanish purebred horses. *Veterinary Record Open*.

[30] Hayama Y., Kobayashi S., Nishida T., Muroga N., Tsutsui T. (2012). Network simulation modeling of equine infectious anemia in the non-racehorse population in Japan. *Preventive Veterinary Medicine*.

[31] Presidente della Repubblica (1954). Regolamento di Polizia Veterinaria.

[32] Ministero della Sanità (1976). Profilassi dell’anemia infettiva degli equini.

[33] Ministero della Salute (2010). Piano di sorveglianza nazionale per l’anemia infettiva degli equidi.

[34] Sala M., Ferri G., Scicluna M. T. (2012). What feedback after five years from the implementation of the Italian National Surveillance Programme (NSP) for equine infectious anemia (EIA). *Journal of Equine Veterinary Science*.

[35] Scicluna M. T., Autorino G. L., Cook S. J., Issel C. J., Cook R. F., Nardini R. (2019). Validation of an immunoblot assay employing an objective reading system and used as a confirmatory test in equine infectious anaemia surveillance programs. *Journal of Virological Methods*.

[36] Nardini R., Autorino G. L., Ricci I. (2016). Validation according to OIE criteria of a monoclonal, recombinant p26-based, serologic competitive enzyme-linked immunosorbent assay as screening method in surveillance programs for the detection of equine infectious anemia virus antibodies. *Journal of Veterinary Diagnostic Investigation*.

[37] Nardini R., Autorino G. L., Issel C. J. (2017). Evaluation of six serological ELISA kits available in Italy as screening tests for equine infectious anaemia surveillance. *BMC Veterinary Research*.

[38] Scicluna M. T., Autorino G. L., Nogarol C. (2018). Validation of an indirect ELISA employing a chimeric recombinant gag and env peptide for the serological diagnosis of equine infectious anemia. *Journal of Virological Methods*.

[39] Ricci I., Nardini R., Rosone F. (2019). Performance and trends of the results of the interlaboratory trials held between 2002–2017 for the serological tests employed for the diagnosis of equine infectious anemia. *Large Animal Review*.

[40] EFSA Panel on Animal Health and Welfare (AHAW), Nielsen S. S., Alvarez J. Assessment of listing and categorisation of animal diseases within the framework of the Animal Health Law (regulation (EU) no 2016/429): infection with Equine herpesvirus-1. *EFSA Journal*.

[41] Maresca C., Scoccia E., Faccenda L., Zema J., Costarelli S. (2012). Equine infectious anemia: active surveillance in central Italy 2007–2009. *Journal of Equine Veterinary Science*.

[42] De Liberato C., Magliano A., Autorino G. L., Di Domenico M., Sala M., Baldacchino F. (2019). Seasonal succession of tabanid species in equine infectious anaemia endemic areas of Italy. *Medical and Veterinary Entomology*.

